# The associations between maternal and child diet quality and child ADHD – findings from a large Norwegian pregnancy cohort study

**DOI:** 10.1186/s12888-021-03130-4

**Published:** 2021-03-08

**Authors:** Tiril Cecilie Borge, Guido Biele, Eleni Papadopoulou, Lene Frost Andersen, Felice Jacka, Merete Eggesbø, Ida Henriette Caspersen, Heidi Aase, Helle Margrete Meltzer, Anne Lise Brantsæter

**Affiliations:** 1 Department of Child Health and Development, Norwegian Institute of Public Health, P.O. Box 222, Skøyen, 0213 Oslo, Norway; 2 Department of Environmental Health, Section of Environmental Exposure and Epidemiology, Norwegian Institute of Public Health, P.O. Box 222, Skøyen, 0213 Oslo, Norway; 3 Department of Nutrition, Institute of Basic Medical Sciences, University of Oslo, P.O. Box 1046, Blindern, 0317 Oslo, Norway; 4 Food & Mood Centre, IMPACT, Deakin University, Geelong, VIC Australia; 5 Centre for Adolescent Health, Murdoch Children’s Research Institute, Parkville, VIC Australia; 6 Black Dog Institute, Randwick, NSW Australia; 7 James Cook University, Townsville, Qld Australia; 8 Division of Infection Control and Environmental Health, Norwegian Institute of Public Health, P.O. Box 222, Skøyen, 0213 Oslo, Norway

**Keywords:** MoBa, Maternal diet quality, Child diet quality, Child ADHD, Bayesian modelling

## Abstract

**Background:**

Attention Deficit Hyperactivity Disorder (ADHD) is a prevalent neurodevelopmental disorder. Effective long-term treatment options are limited, which warrants increased focus on potential modifiable risk factors. The aim of this study was to investigate associations between maternal diet quality during pregnancy and child diet quality and child ADHD symptoms and ADHD diagnosis.

**Methods:**

This study is based on the Norwegian Mother, Father and Child Cohort Study (MoBa). We assessed maternal diet quality with the Prenatal Diet Quality Index (PDQI) and Ultra-Processed Food Index (UPFI) around mid-gestation, and child diet quality using the Diet Quality Index (CDQI) at 3 years. ADHD symptoms were assessed at child age 8 years using the Parent Rating Scale for Disruptive Behaviour Disorders. ADHD diagnoses were retrieved from the Norwegian Patient Registry.

**Results:**

In total, 77,768 mother-child pairs were eligible for studying ADHD diagnoses and 37,787 for ADHD symptoms. Means (SD) for the PDQI, UPFI and CDQI were 83.1 (9.3), 31.8 (9.7) and 60.3 (10.6), respectively. Mean (SD) ADHD symptom score was 8.4 (7.1) and ADHD diagnosis prevalence was 2.9% (male to female ratio 2.6:1). For one SD increase in maternal diet index scores, we saw a change in mean (percent) ADHD symptom score of − 0.28 (− 3.3%) (CI: − 0.41, − 0.14 (− 4.8, − 1.6%)) for PDQI scores and 0.25 (+ 3.0%) (CI: 0.13, 0.38 (1.5, 4.5%)) for UPFI scores. A one SD increase in PDQI score was associated with a relative risk of ADHD diagnosis of 0.87 (CI: 0.79, 0.97). We found no reliable associations with either outcomes for the CDQI, and no reliable change in risk of ADHD diagnosis for the UPFI.

**Conclusions:**

We provide evidence that overall maternal diet quality during pregnancy is associated with a small decrease in ADHD symptom score at 8 years and lower risk for ADHD diagnosis, with more robust findings for the latter outcome. Consumption of ultra-processed foods was only associated with increased ADHD symptom score of similar magnitude as for overall maternal diet quality, and we found no associations between child diet quality and either outcome. No causal inferences should be made based on these results, due to potential unmeasured confounding.

**Supplementary Information:**

The online version contains supplementary material available at 10.1186/s12888-021-03130-4.

## Background

The World Health Organization (WHO) defines neurodevelopmental disorders as one of today’s greatest public health challenges [1], with Attention Deficit Hyperactivity Disorder (ADHD) being one of the most prevalent among children in Norway and worldwide [2, 3]. ADHD is characterized by a number of behavioural traits, including inattention, impulsivity and hyperactivity [4] and it typically co-occurs with delayed language development, motor functions, impaired emotional control as well as with other psychiatric disorders [5, 6]. Furthermore, impairments related to ADHD often persist into adulthood [7–10], alongside poorer social functioning and an increased risk of unemployment [3, 6].

ADHD aetiology is multifactorial and complex [6], involving a genetic contribution [6, 11], as well as social- and environmental factors [12]. Because treatment options available for young children are limited [13, 14] and due to the relatively poor long-term effectiveness of medical treatment for ADHD [15], identification of early-life modifiable risk factors, such as nutritional factors, could be a key strategy in improving prevention approaches for ADHD in children [13, 16].

Findings from investigations into associations between child diet and ADHD have been equivocal. Some research on ADHD patients have found suboptimal levels of several nutrients, including zinc [17], iron [18], magnesium [19] and Omega-3 [18, 20], but dietary interventions aiming to reduce symptoms have yielded mixed results [21, 22]. A recent meta-analysis reviewed the scarce literature investigating dietary patterns and ADHD diagnosis or symptoms in children [23]. Overall, the authors found higher risk of ADHD diagnosis or symptoms for children having low scores on a healthy dietary pattern and high scores on an unhealthy dietary pattern. However, all studies were observational, where half investigated associations cross-sectionally, which severely impedes a causal interpretation of these associations.

It has been suggested that an optimal maternal diet during pregnancy might be even more crucial to child ADHD outcomes than early childhood diet [15]. Surprisingly, however, the possible impact of maternal diet quality during pregnancy on child neurodevelopmental functions related to ADHD has been largely neglected until recently. The increased interest in this area is likely due to the evidence that the in-utero environment might be vital in the development of neurodevelopmental disorders [24–26]. Research on the effects of severe and prolonged maternal malnourishment on brain development in offspring highlights the important role of maternal diet [18], particularly during the prenatal and early postnatal period [6]. It remains less clear how subtle differences in maternal diet quality during pregnancy might influence the trajectory of child neurodevelopmental disorders.

We have not identified any research investigating overall maternal diet quality during pregnancy in relation to child ADHD diagnosis. Moreover, the limited amount of literature investigating prenatal and child diet in relation to neurodevelopmental problems associated with ADHD has indicated that there exists a possible association [27, 28]. However, these associations tend to be either small or from smaller studies [29]. Hence, there is a need for large population-based studies that investigate longitudinal associations between prenatal and early postnatal diet quality and ADHD.

The aim of this paper is to investigate the associations between both maternal diet quality during pregnancy and child diet quality, using diet quality indices, and maternally reported child ADHD symptoms at 8 years and child ADHD diagnosis retrieved from the Norwegian Patient Registry.

## Methods

We followed the Strengthening the Reporting of Observational studies in Epidemiology (STROBE) statement as the reporting guideline [30] (see Additional file 1).

### Study population and design

**Fig. 1:**
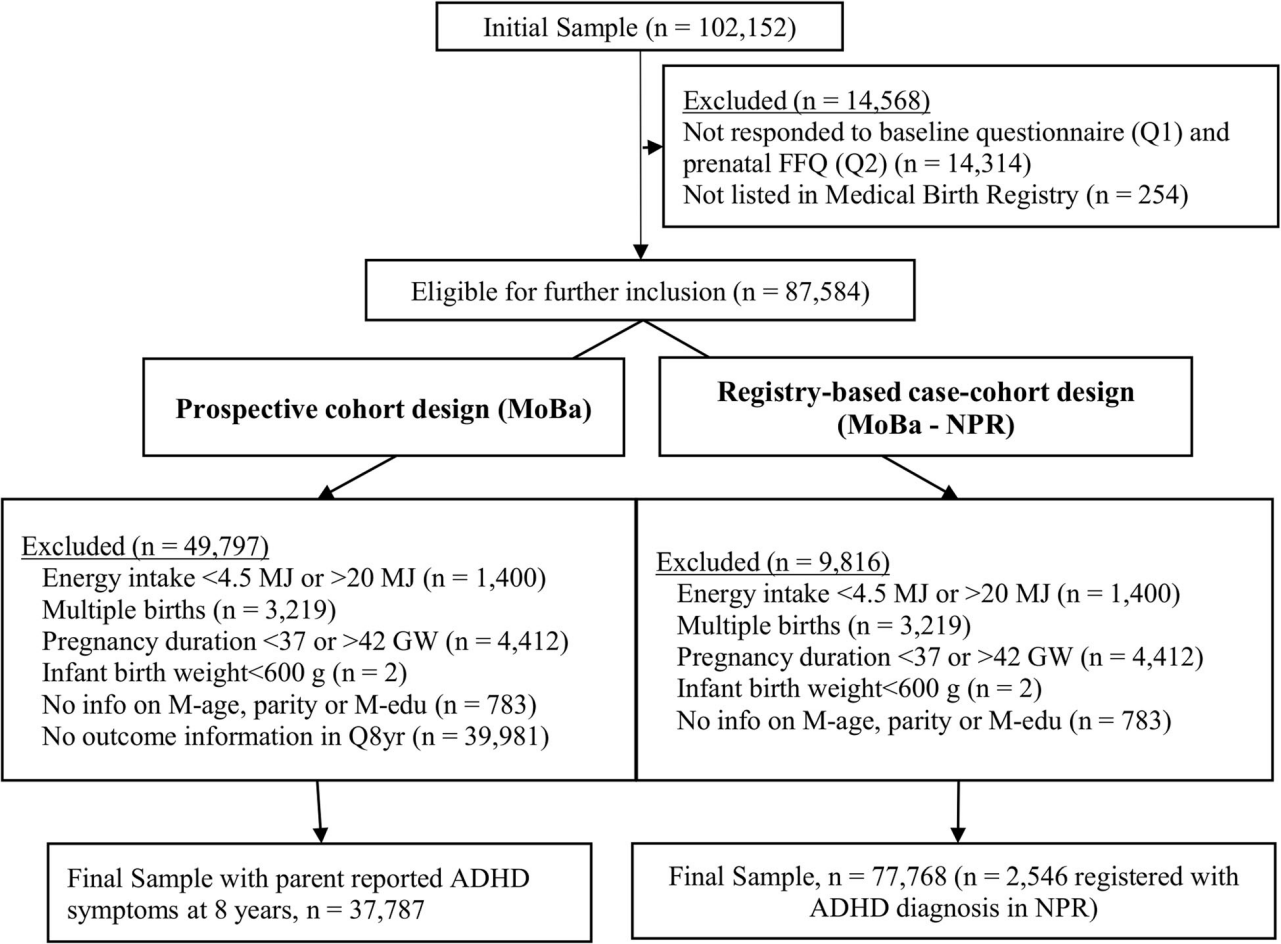
Flow chart of inclusion and exclusion criteria and final selection of Norwegian Mother, Father and Child Cohort Study (MoBa) participants (FFQ: food frequency questionnaire introduced in 2002; NPR: Norwegian Patient Registry; GW: gestational week; M-age: maternal age; M-edu: maternal education; Q8yr: MoBa questionnaire at child age 8 years)

The Norwegian Mother, Father and Child Cohort Study (MoBa) is an ongoing prospective population-based pregnancy cohort study conducted by the Norwegian Institute of Public Health, aiming to investigate how genetic- and environmental factors affect health outcomes [31]. Pregnant women were recruited across Norway between 1999 and 2008, prior to the routine ultrasound screening around gestational week (GW) 18, with 41% of the invited women consenting to participate. The cohort includes 114,500 children, 95,200 mothers and 75,200 fathers. The participating pregnant women answered questionnaires at regular intervals prenatally (GW 22 and 30) and during the postnatal phase (6 months, 18 months, and 3, 5, 7, and 8 years after birth). The response rates for the three prenatal questionnaires were between 91 and 95% [31]. In this study we use data from the two MoBa questionnaires answered in pregnancy (baseline questionnaire and FFQ) and the questionnaires at child age 3 and 8 years. We based this study on version 11 of the quality-assured data files released for research on 18 October 2018, which provided us with an initial sample of *n* = 102,152 mother-child dyads. This data was linked with data from the Norwegian Medical Birth Registry (MBRN) and with the Norwegian Patient Registry (NPR), a national administrative health registry with patient information reported from hospitals and outpatient clinics in Norway. We utilised two different study designs in this paper; a prospective cohort design and a registry-based case cohort design. Figure 1 describes the process of participant inclusion for both study designs.

### Exposure definition

#### Maternal diet quality during pregnancy

The MoBa food frequency questionnaire (FFQ) was administered around gestational week 22 and provides comprehensive information about maternal dietary habits and intake of foods, beverages and dietary supplements since beginning of pregnancy [32]. The MoBa FFQ was specifically developed for the MoBa [33] and has been validated using biomarkers in urine and blood samples in addition to a 4-day weighed food diary [33–36]. We converted intake frequencies to intake in grams per day for 255 foods and beverages assuming standard portion sizes and using FoodCalc [37], and the Norwegian food composition database [38].

We evaluated maternal diet quality during pregnancy by applying two dietary indices to the FFQ-derived data. The first index, called prenatal diet quality index (PDQI) (possible range of scores = 0–110), captures adherence to the Norwegian food-based dietary guidelines. We have previously used this index to investigate associations with child developmental outcomes [29]. The second index, called the ultra-processed food index (UPFI) (possible range = 0–100%), captures the relative contribution of ultra-processed foods to total energy intake. The UPFI was used to measure the opposite aspects of diet quality to those captured by the PDQI.

The PDQI is largely based on the well-known Healthy Eating Index [39]. We calculated the PDQI score using the method put forward by von Ruesten et al. [40] with modifications to correspond to the current Norwegian food-based dietary guidelines [41]. We list an overview of the components in Table 1. Detailed description of the calculation has been published in a previous article [29] and is included in Additional file 2.

**Table 1:** Overview of the PDQI components, corresponding recommended intake and maximum component scores

PDQI components	Recommended intake	Maximum component score*
Fresh fruits & berries	Minimum 250 g/day	10
Vegetables	Minimum 250 g/day	10
Whole grain	Ca. 70 g/day for women	10
Total Fish	300–450 g/week	5
Fatty fish	Minimum 200 (to max. 450) grams/week	5
Red meat	Maximum 500 g/week	10
Dairy	3 servings (1 serving = 20 g cheese or 2 dl milk or 1 pot yoghurt (125 g)	10
Saturated fat	Maximum 10% of total energy	10
Trans fat	Maximum 1% of total energy	10
Salt	Maximum 6 g salt/day (= 2.4 g sodium/day)	10
Added sugar	Maximum 10% of total energy intake	10
Dietary diversity score	Measure of diversity of foods within 4 major food groups eaten daily (grains, vegetables, fruits, dairy)	10

PDQI: Prenatal Diet Quality Index.

*Maximum total score = 110

The second maternal dietary index, the UPFI, was created by calculating the relative contribution of energy from ultra-processed foods to total energy intake. Following the NOVA classification [42], foods and beverages are classified into four distinct categories according to the level of processing, ranging from minimally processed (group 1, e.g. fresh fruits and vegetables, raw nuts, legumes, whole meats with no additives or preservatives), to ultra-processed (group 4, e.g. savoury and sweet snacks, breads containing emulsifiers, reconstituted meat products, products with artificial sweeteners). We only included foods from group 4, as our aim was to investigate ultra-processed foods and their contribution to the overall energy intake, and we already include a measure of overall diet quality represented by the PDQI. For a full list of the food and beverage groups included in the UPFI, see Additional file 3. We calculated and summed the energy intake from the MoBa FFQ items in group 4 and calculated its relative contribution to total energy intake to create the UPFI score, with possible range of 0–100, corresponding to percent contribution.

#### Child diet at 3 years

Child intake of 36 food and beverage items was assessed by parent-reported food intake questions included in the MoBa questionnaire at 3 years. We evaluated overall child diet quality by defining the Diet Quality Index (DQI), as developed by Huybrechts et al. for use in pre-school children [43], which also has been used in a multi-country study of more than 7000 European pre-schoolers [44]. We refer to it as child diet quality index (CDQI) throughout the paper to make a clear distinction from the PDQI. In common with the PDQI, the CDQI assesses adherence to dietary recommendations. As basis for the calculations, we used information from the food intake questions included in the MoBa questionnaire at 3 years, where mothers report the child’s average intake frequency of 36 selected foods and beverages covering 6 main food groups (FG’s) (dairy, fruits, vegetables, meat, fish, grains) in addition to sweets/snacks. Since there was no assessment of water intake in the MoBa questionnaire at 3 years, we used reported water intake from the MoBa questionnaire at 18 months as proxy for water intake at 3 years. Intake frequencies were converted to intakes in grams per day by using standardized portion sizes [45, 46].

The CDQI consists of the following components: diet diversity, diet quality and diet equilibrium, where the total CDQI score is the average score across the three components, with higher scores equalling better diet quality*.* The original DQI also includes a meal pattern component, but there were no questions on child meal patterns in the MoBa questionnaires. Below is a brief description of each component. For more detailed information on the calculation method, see Additional file 4 and Huybrechts et al. [43].

The diet diversity component (possible range of 0–100%), reflects the intake variation in the foods by giving one point if the child had consumed at least one daily serving from each main FG.

The diet quality component (possible range of − 100 - 100%) is used to categorize food intake, within each FG, according to their quality, into preferred foods (e.g. High fibre bread in the bread and cereal group) factored by 1, moderation foods (e.g. low-fibre bread in the bread and cereal group) factored by 0 and the “low nutrient, energy dense” foods (e.g. sweet bread/buns in the bread and cereal group); factored by − 1. After categorization based on intake in grams per day, the foods were summed and divided by the total food intake.

The diet equilibrium consists of adequacy and moderation (possible range of 0–100%). The diet equilibrium component introduces the concept of dietary balance to the CDQI, taking into account both “moderation” and “adequacy”: intake moderation (percentage of intake exceeding the upper level of the recommendation), with particular focus on limiting the intake of low nutrient and energy dense foods, subtracted from intake adequacy of foods in the preferred group (percentage, reported intake of food/minimum recommended intake), divided by total number of FGs.

### Outcome definitions

#### ADHD symptom score - MoBa

Child ADHD symptoms were assessed in the MoBa eight-year questionnaire, using items from the Parent Rating Scale for Disruptive Behaviour Disorders [47]. Mothers reported on child ADHD symptoms on a four-point Likert scale (never/rarely, sometimes, often, or very often, scored 0–3) covering two domains: inattention (nine items) and hyperactivity/impulsivity (nine items). We calculated ADHD symptom sum scores, with higher scores indicating more and stronger symptoms.

#### ADHD diagnoses - NPR

We obtained information about children’s ADHD diagnoses from the Norwegian Patient Registry (NPR). The NPR originated in 1997, and person-identifiable records are available from 2008 onwards [48]. The NPR classifies an ADHD diagnosis according to the International Classification of Disease 10th revision (ICD-10) as hyperkinetic disorder [49]. Diagnostic information from the NPR was retrieved and linked to the MoBa data file, comprising all MoBa children registered at least once with an ICD-10 code of hyperkinetic disorder (coded as F90.0, F90.1, F90.8, or F90.9), corresponding to an ADHD diagnosis, between 2008 and 2017. Hyperkinetic disorder requires the combination of persistent inattentive and hyperactive-impulsive symptoms before the age of six and impairment in two or more settings.

#### Covariates

We assessed a number of covariates for inclusion in the analyses, based on previous knowledge and directed acyclic graphs (DAGs) [50]. For maternal diet quality during pregnancy, the final covariate set comprised: maternal pre-pregnancy BMI in kg/m^2^; maternal education (< 12 years, Upper secondary (12 years), Bachelor (15 years), Master + (17+ years)); smoking during pregnancy (yes/no); alcohol intake during pregnancy (yes/no); reported symptom score of maternal prenatal depression (0–3) and ADHD (0–4); maternal age (in years); child sex (boy/girl); parity (0–3); child diet quality score at 3 years (CDQI) and child season of birth (in quarters) (see Additional file 5 for DAG). We adjusted the maternal diet models for child diet as this removes bias from common unobserved causes of maternal and child diet.

For child diet quality, the final covariate set comprised: maternal pre-pregnancy BMI in kg/m^2^; maternal education (< 12 years, Upper secondary (12 years), Bachelor (15 years), Master + (17+ years)); maternal symptoms of ADHD (0–4); maternal age (in years); maternal diet quality score during pregnancy (PDQI); child sex (boy/girl); parity (0–3); reported child sleep problems at 3 years (yes/no); and child season of birth (in quarters) (see Additional file 6 for DAG). The maternal variables included, except for ADHD symptoms, were assessed during pregnancy. Lastly, we included birth quarter as a covariate for all exposure variables to increase the precision of our estimates, as children born in October–December are 45% more likely to have an ADHD diagnosis compared to children born in January–March [51].

### Statistical analyses

Reliability and validity of the PDQI in the MoBa sample have been reported previously [29]. We evaluate construct validity for the UPFI, and CDQI construct validity and reliability. For construct validity we calculate differences between groups known to have different diet quality and report the differences as Hedge’s g. Hedge’s g is calculated by dividing the mean differences of two groups by their pooled standard deviation and is expressed in units of the pooled standard deviation. E.g. a Hedge’s g of 0.5 indicate that there is a difference in means between groups of 0.5 pooled standard deviation. For reliability we report the Omega total [52], which is an index of internal consistency, i.e. how well the items included in a scale (e.g. the diet diversity, quality and equilibrium components in the CDQI) measure one underlying construct, i.e. the CDQI. The Omega is estimated using a factor analysis with oblique rotation, followed by a Schmid-Leiman transformation for general factor extraction [52], using the Omega function in the Psych R package [53].

We evaluated the strength of associations between the diet quality indices and outcomes by estimating Bayesian regression models, using a beta-binomial likelihood for ADHD symptoms and logistic regression for ADHD diagnosis. For the analyses we utilised the R statistical software, version 3.4.3 [54] and the brms package, version 2.1.0 [55]. To avoid losing variation in our data, all exposure and outcome variables except for ADHD diagnosis were kept in its continuous form, in line with suggested approaches [56].

For all exposure variables, we used child sex and maternal ADHD symptoms as stratification variables to explore possible differences in associations between strata. MoBa assesses maternal ADHD symptoms in the 3 year questionnaire with the Adult ADHD Self-Report Scale (ASRS), which consists of six questions relating to symptoms of adult ADHD based on the DSM-IV diagnosis criteria [57]. Stratifying on maternal ADHD symptoms also adjusts for maternal ADHD as a confounder that influences maternal diet and child ADHD.

For child ADHD symptoms as outcome, we report the results as average marginal effect (AME), i.e. the average change in the outcome score for a one standard deviation (1SD) change in the exposure. We report both change in mean score (absolute AME) and corresponding percent change (relative AME) with corresponding credible intervals (CI’s) (CI’s can be considered as synonymous with confidence intervals in traditional statistical approaches). For ADHD diagnosis, we report the results as relative risk (RR) with corresponding CI’s. For each exposure, we fit two separate models; one crude and one adjusted including all covariates.

Based on prior predictive simulations, we set the prior for population level (fixed) effects to a normal distribution with a mean of 0 and a standard deviation of 2, and the prior for the standard deviation of random effects to a half-normal distribution with a mean of 0 and a standard deviation of 2 [58]. Note that priors will not have substantial influence on the final estimates, due to the large sample size of MoBa [59, 60].

Participation and loss to follow-up in MoBa is not random but dependent on maternal education, age and child parity, which can lead to biased estimates [61]. Therefore, we use inverse probability weighting (IPW) based on these three variables to control for bias due to self-selection into the study and cohort attrition [61, 62]. We calculated weights from simple participation probabilities, i.e. the number of mothers in a population subgroup in the study sample divided by the number of mothers in the same subgroup in the target population (mothers who gave birth in Norway during MoBa’s inclusion period). We obtained population data from Statistics Norway, which provided maternal age, parity and education for the Norwegian pregnant population for the MoBa recruitment period.

For all models we used complete cases for the variables included in each respective model, see Table 2 for details. We believe using complete case analyses is justified due to the uncertainties relating to the pattern of missingness in our data and since we use IPW in all analyses.

**Table 2:** Complete cases for variables included in each model for all exposures and outcomes

	ADHD symptoms		ADHD diagnosis (cases in parenthesis)	
	Crude model	Adjusted model	Crude model	Adjusted model
PDQI/ UPFI	*n* = 31,152	*n* = 19,403	*n* = 46,976 (*n* = 1412)	n = 27,769 (*n* = 812)
CDQI	*n* = 22,699	n = 22,290	*n* = 32,687 (*n* = 956)	n = 32,034 (*n* = 936)

## Results

### Reliability and construct validity

#### UPFI and PDQI

We investigated whether the UPFI was able to differentiate between groups known to have different diet quality: smokers vs. non-smokers, lower (< 12 years education) vs higher (master degree) education, younger mothers (<=21 years) vs older mothers (> = 30 years) and people presenting with and without depressive symptoms. We calculated standardized mean differences between groups, reported as Hedges’ g, with a medium effect size seen for low vs. high education (g = 0.59), low vs. high age (g = 0.48) and smoking vs. no smoking (g = 0.53) and small effect size for with vs. without depressive symptoms (g = 0.24). In addition, the UPFI score correlated with energy adjusted intakes of key nutrients in the FFQ in the expected direction (fibre (r = − 0.50), sugar (r = 0.59), protein (r = − 0.55) saturated fat (r = 0.30)), as well as with the PDQI (r = − 0.53). In a recent paper [29], we described reliability and construct validity for the PDQI.

#### CDQI

Reliability analysis revealed an Omega total of 0.69, which is satisfactory. We also explored how well the CDQI was able to differentiate between groups related to the mother (age, education and diet quality during pregnancy) and the child (screen time and physical activity) known to have different diet quality. We calculated standardized mean difference between groups, reported as Hedges g. We saw a large effect size for maternal diet quality during pregnancy (g = − 0.93 (<=20th percentile vs. > = 80th percentile)) and screen time at 3 years (g = − 0.79 (> = 3 h/day vs. < 1 h/day)), medium effect size for maternal education (g = − 0.64 (< 12 years education vs. master degree)) and child outdoor time at 3 years (g = − 0.55 (< 1 h/day vs. > 3 h/day) and small effect size for maternal age (g = − 0.35 (<=21 years vs. > = 30 years)). Also, the CDQI showed correlations with the maternal dietary indices in the expected direction (PDQI: r = 0.32, UPFI: r = − 0.24).

### Main results

We saw a strong relationship between ADHD symptoms in the MoBa 8-year questionnaire and ADHD diagnosis, as the median ADHD symptom score of children with a registered diagnosis was + 2.8 SD higher than the score of those without a registered diagnosis.

The mean (max) PDQI, UPFI and CDQI scores were 83.1 (107.1), 31.8 (89.5) and 60.3 (86.5), respectively. The mothers were on average 30.2 years of age at delivery, with 67% having at least a bachelor’s degree. The mean (range) age when the children were first registered with an ADHD diagnosis in NPR was 8 (2.6–13.8) years, with 80% being 6 years old or older. For additional details on descriptive statistics, see Table 3.

**Table 3:** Descriptive Statistics for the Exposures, Outcomes and Covariates^*^

Variables	n	Missing		Mean (SD)	Median (IQR)	Range
		n	%			
**Exposures**						
PDQI score	77,768	0	0%	83.1 (9.3)	83.9 (12.7)	38.7–107.1
UPFI score	77,768	0	0%	31.8 (9.7)	31.2 (12.7)	0–89.5
CDQI score	32,937	44,831	58%	60.3 (10.6)	61.5 (13.9)	2.8–86.5
**Outcomes**						
ADHD symptom score	37,796	39,972	51%	8.4 (7.1)	7 (7)	0–54
ADHD diagnosis, NPR		0	0%			
*Yes*	2546					
*No*	75,222					
**Covariates**						
Maternal age	77,768	0	0%	30.2 (4.5)	30 (6)	15–47
Pre-pregnant BMI	75,804	1964	3%	24.0 (4.3)	23.1 (4.8)	12.5–57.8
Maternal ADHD symptom score, mean	46,976	30,792	40%	1.1 (0.57)	1 (0.83)	0–4
Maternal Hopkins symptom score, mean	76,321	1447	2%	0.25 (0.39)	0 (0.4)	0–3
Maternal completed education		0	0%			
*< 12 years*	5474					
*Upper secondary*	21,234					
*Bachelor*	32,497					
*Master*	18,563					
Prenatal smoking		1099	1%			
*Yes*	5816					
*No*	70,853					
Prenatal energy intake (MJ)	77,768	0	0%	9.7 (2.6)	9.4 (3.2)	4.5–20
Parity		0	0%	0.8 (0.8)	1 (1)	0–3
*0*	35,140					
*1*	27,922					
*2*	11,634					
*3*	3072					
Child sex		0	0%			
*Girls*	38,037					
*Boys*	39,731					
Child sleep problems at 3 y		29,594	38%			
*Yes*	2648					
*No*	45,526					

^*^For categorical and dichotomous variables, only n is given

**Fig. 2:**
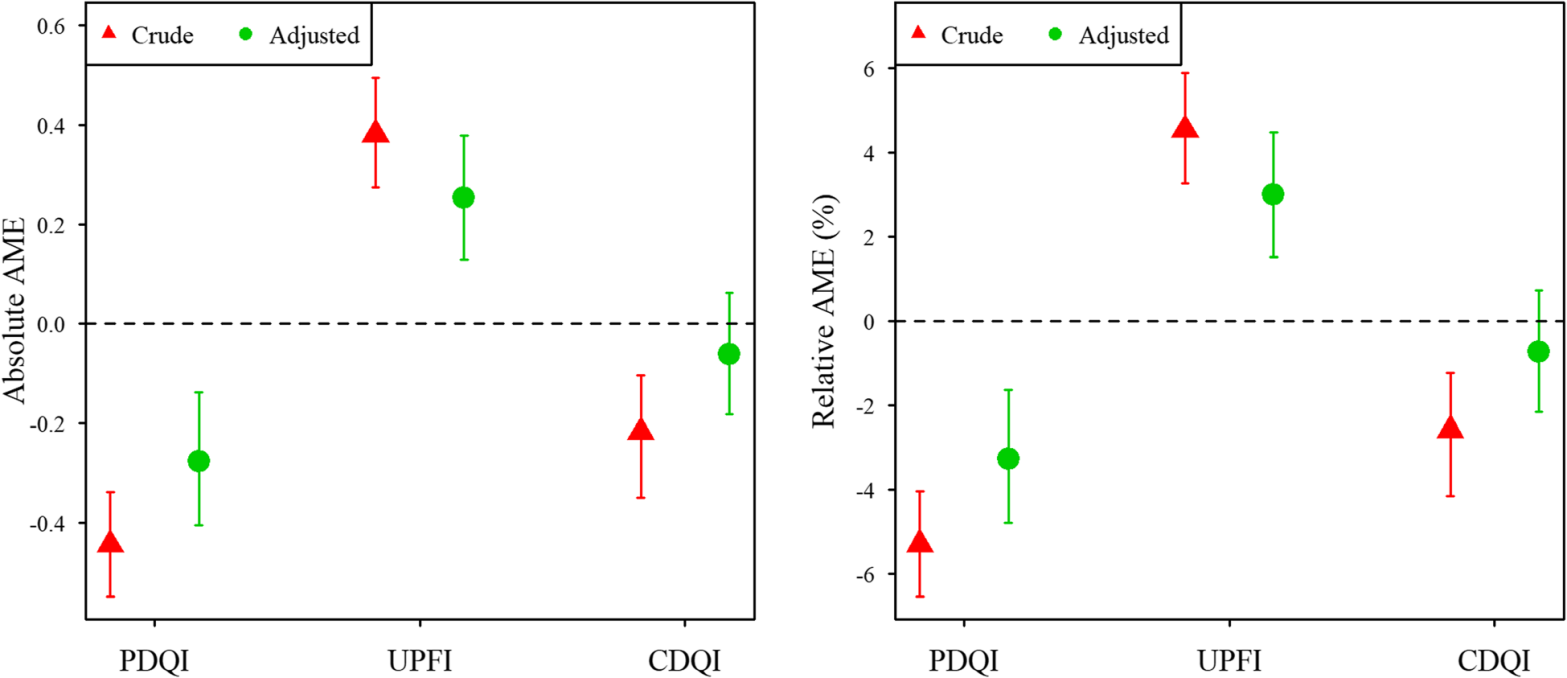
Absolute AME and corresponding relative AME for ADHD symptom score at 8 years, for a 1SD increase in PDQI, UPFI and CDQI score (Covariates in adjusted models: For PDQI and UPFI: maternal pre-pregnancy BMI, maternal education, smoking and alcohol intake during pregnancy, maternal symptoms of depression and ADHD, maternal age, parity, child sex, child diet and child birth quarter. For CDQI: maternal pre-pregnancy BMI, maternal education, maternal symptoms of ADHD, maternal age, prenatal diet quality, child sex, parity, child sleep problems (3y) and child birth quarter)

For a one standard deviation (1SD) increase in mean diet quality score, we obtained the following results for ADHD symptom score in the adjusted models: a change in mean ADHD symptom score of − 0.28 (CI: −0.41, −0.14) for the PDQI and + 0.25 (CI: 0.13, 0.38) for the UPFI, corresponding to a relative change in mean ADHD symptom score of −3.3% (CI: −4.8, −1.6%) and + 3% (CI: 1.5, 4.5%), respectively (Fig. 2 and Additional file 7). These changes are equivalent to a 1SD change in the PDQI and UPFI scores being associated with a − 0.04 SD and + 0.035 SD change of the ADHD symptom score, respectively. We found no change in ADHD symptom score in the adjusted model for CDQI (− 0.7% (CI: − 2.2, 0.7%)).

**Fig. 3:**
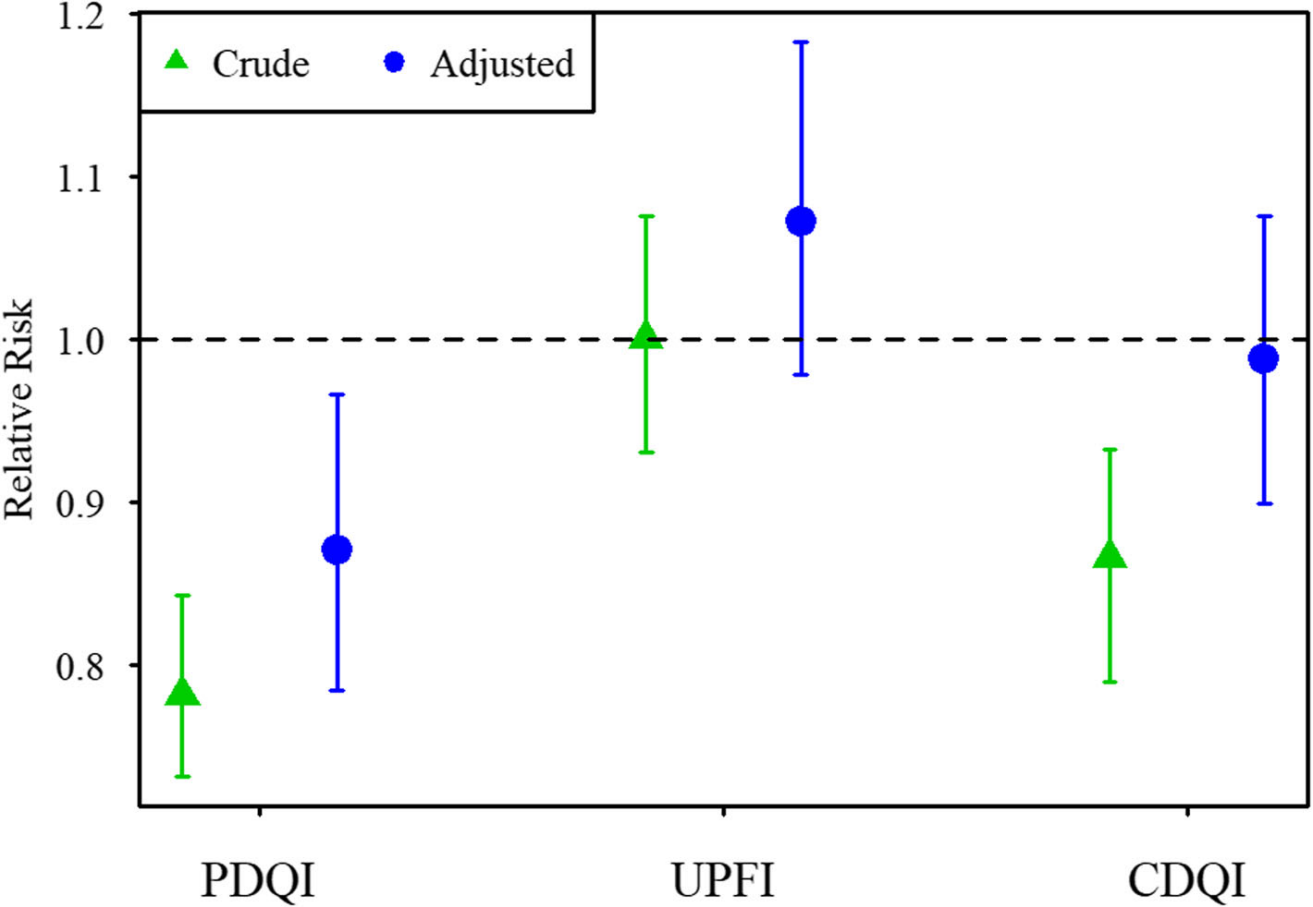
Relative risk of ADHD diagnosis, for 1SD increase in PDQI, UPFI and CDQI score (Covariates in adjusted models: For PDQI and UPFI: maternal pre-pregnancy BMI, maternal education, smoking and alcohol intake during pregnancy, maternal symptoms of depression and ADHD, maternal age, parity, child sex, child diet and child birth quarter. For CDQI: maternal pre-pregnancy BMI, maternal education, maternal symptoms of ADHD, maternal age, prenatal diet quality, child sex, parity, child sleep problems (3y) and child birth quarter)

For ADHD diagnosis, we found a relative risk in the adjusted models of 0.87 (CI: 0.79, 0.97) for the PDQI. To compare, for a 1SD increase in ADHD symptom score, the relative risk of ADHD diagnosis is 3.3 (CI: 3.0, 3.7). We found no reliable change in risk for the UPFI (1.07 (CI: 0.99, 1.18)) or CDQI (0.99 (CI: 0.90, 1.08) (Fig. 3 and Additional file 8). Analyses stratified on child sex and maternal ADHD symptoms revealed no differences in associations for either ADHD symptom scores (see Additional file 9) or ADHD diagnosis (see Additional file 10) between strata.

**Fig. 4:**
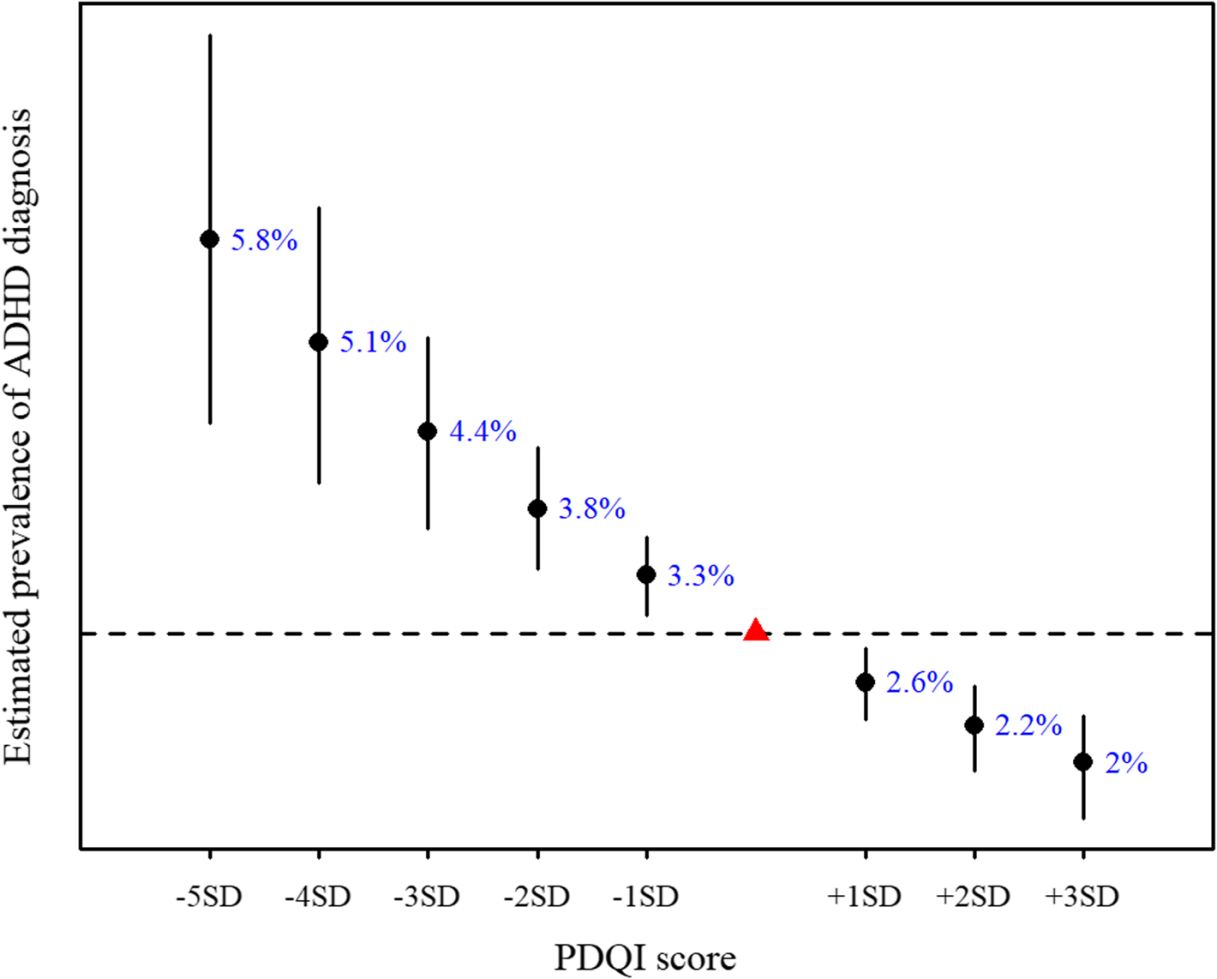
Estimated prevalence of ADHD diagnosis in MoBa children with 1SD incremental change in mean PDQI score, from -5SD to +3SD from the mean PDQI score of the study sample (red triangle). Based on fully adjusted model with complete cases and IPW (total *n* = 27,769)

We estimated changes in prevalence of ADHD diagnosis for incremental changes in the PDQI score from the adjusted model (Fig. 4). To include PDQI scores covering the observed range of scores in the study population (38.7–107.1), we calculated change in prevalence of ADHD diagnosis for -5SD (PDQI score of 37) to +3SD (PDQI score of 111) from the mean PDQI score. In the effective study sample, the number of children with an ADHD diagnosis was 812, corresponding to a prevalence of 2.9%, represented by the triangle on the dashed line in Fig. 4. For example, the estimated prevalence of child ADHD in a population of mothers with a mean PDQI score 3SD’s below the study population mean is 4.4%, i.e. a 52% increase in prevalence.

## Discussion

In this study, we investigated the associations between maternal and child diet quality and maternally reported ADHD symptom score at 8 years and child ADHD diagnosis. We observed inverse associations between maternal diet quality during pregnancy (measured by the PDQI) and both child ADHD symptom scores and child ADHD diagnosis, and a positive association between the contribution of ultra-processed food to total maternal energy intake in pregnancy (measured by the UPFI) and child ADHD symptom scores. We found no reliable association between UPFI and child ADHD diagnosis, or between child diet quality (measured by the CDQI at 3 years) and either outcome.

The difference in results for maternal diet quality during pregnancy and child diet quality at 3 years might be due to difference in measurement detail, since the maternal FFQ is more extensive than the questions about food intake in the child questionnaire (255 vs. 36 food items), which is likely to influence the specificity and accuracy of the instrument and subsequent dietary index scores calculated from those instruments. Nevertheless, we acknowledge that the child dietary questions include the main foods and beverages consumed at three years of age.

If the associations between maternal diet quality during pregnancy and child diet quality and ADHD symptoms and diagnosis are due to causal effects from maternal diet quality on child development, these results would support the idea of prenatal programming. During critical stages of the prenatal period, the foetus may be particularly sensitive to environmental influences like maternal nutrition, and the subsequent insults based on these influences may sustain long after birth and into adulthood [26]. A proposed mechanism behind prenatal programming is epigenetic modifications [25, 63–65] via e.g. immune activation [24, 66, 67]. Interestingly, one study has found an association between prenatal high fat/high sugar diet and ADHD in youth with early onset conduct problems indirectly via epigenetic modifications of the insulin-like growth factor gene [68]. Still, studies that use robust causal identification strategies to ensure that observed associations between diet and ADHD are due to causal effects of diet are an important next step.

We found no associations between child diet quality at 3 years and either outcome, which adds to the so far undecided evidence base investigating child diet and ADHD. A recent meta-analysis found healthy dietary patterns to be associated with low ADHD symptoms in children (3–11 years old) and adolescents (12–16 years old) and unhealthy dietary patterns a risk factor for ADHD, but the authors emphasize that due to limitations in study design across most included studies, these associations do not constitute evidence for causal effects [23]. Also, the authors do not explain how they dealt with dependencies in the effect estimates, nor if they investigated for sources of heterogeneity other than publication bias, which indicates that further caution should be made in making firm inferences from their findings. Conversely, Mian et al. [69] found ADHD symptoms at 6 years to be predictive of child diet quality assessed at 8 years, but not vice versa, with ADHD symptoms at 10 years, suggesting that postnatal diet quality does not influence ADHD symptoms, but rather that some ADHD symptoms, e.g. low levels of impulse control, may result in a poorer diet.

We found associations of similar magnitude for both maternal diet quality indices in relation to child ADHD symptom score, however the associations were small. This is in line with the small association estimates found in a previous review and meta-analysis investigating maternal diet quality and child neurodevelopmental outcomes. More recently, Mesirow and colleagues [70] studied aspects of maternal diet quality in relation to child behavioural issues in children with either low conduct problems or early-onset persistent conduct problems. They found that, specifically for children presenting with early-onset conduct problems, mothers had a poorer diet quality (lower fish intake, higher processed food consumption) compared to mothers with children having low conduct problems. Additionally, maternal processed food consumption was associated with higher childhood hyperactivity (4–10 years) independent of conduct problem trajectories, but the effect estimate was small.

Isaac and Oates [71] propose that large effect estimates cannot be expected in generally healthy populations with adequate diet quality, such as the MoBa sample which consists of, on average, well educated mothers in a rich, industrialized country, when investigating outcomes related to child developmental functions. Isaac and Oates further argue that even though results might indicate an association, there might be no clinical significance as the absolute effect estimates might be minute [71]. This increases our confidence in proposing that associations seen between the maternal diet quality indices and child ADHD symptoms in generally healthy populations with, on average, adequate diet quality are so small that they have no clinical relevance. However, small effects have the potential to influence outcomes at the population level, particularly in the situation where the exposure, in this case diet, affects the entire population. Still, more research is needed to understand the association between maternal diet and child ADHD symptoms and diagnoses in populations with a higher prevalence of inadequate maternal diet quality during pregnancy.

For child ADHD diagnosis, we only found associations with the PDQI (indicates adherence to the Norwegian food-based dietary guidelines) and not for the UPFI (indicates consumption of ultra-processed foods). It is likely that the ADHD diagnosis outcome (retrieved from patient registry) is less afflicted by bias than the ADHD symptom score outcome (based on parent reported symptoms), which might be one reason for the difference in results for the two outcomes. If we assume that the results for ADHD diagnosis are more accurate due to less presence of bias, compared to the associations seen for the ADHD symptom score outcome, it can be an indication of overall maternal high diet quality during pregnancy being more important for ADHD development than the proportion of intake of ultra-processed foods, which are mostly of low nutritional value. This is in line with recent findings investigating maternal dietary patterns and birth outcomes, which found stronger associations with preterm birth for the healthy pattern compared to the unhealthy, and the opposite trend for birth weight [72], but only for data driven patterns, and not a-priori defined indices that are more generalizable. Looking at studies with outcomes that are related to ADHD, Jacka et al. [73] found the opposite trend, wherein the unhealthy dietary pattern showed a stronger association with symptoms of child behavioural difficulties compared to the healthy dietary pattern. Conversely, Steenweg-de Graaff et al. [74] found similar strengths of associations for low adherence to a healthy dietary pattern and high adherence to an unhealthy dietary pattern in relation to child externalizing difficulties.

In sum, the evidence so far relating to the comparison of association strengths between healthy and unhealthy diets is equivocal. Perhaps the most plausible reason for this is due to heterogeneous methodological approaches related to the dietary definitions and statistical analyses chosen, which should serve as an encouragement to develop standardized methodological approaches within the nutritional epidemiological field.

### Strengths and limitations

The strengths of this study include a large sample size, validation of dietary indices used and use of IPW to account for selection bias. We use robust statistical methods that to our knowledge have not been previously used within the nutritional epidemiology field. Also, investigations of associations between prenatal diet quality and child ADHD diagnosis has not previously been investigated.

One limitation of this study is that self-reported information, particularly in relation to aspects of health, introduces many challenges [75–77], and collecting data with FFQ’s has generated much criticism [78]. However, the maternal FFQ utilized in this study has been extensively validated and was explicitly developed for the target population [34]. Moreover, using a composite measure of overall diet quality is more robust than looking at e.g. estimations of single nutrients with regards to misreporting, and it is a recommended method for investigating diet-disease relationships [79].

Another limitation is that the MoBa is a selected group of participants and not representative of the whole population of Norwegian mothers and children. On average, MoBa mothers are older and more educated than the general pregnant population in Norway [80], and both these factors are related to better prenatal diet quality and lower levels of child difficulties. However, as we used IPW for maternal age, education and child parity in our analyses, the results might be generalized with some caution, to populations similar to MoBa’s source population.

There are some limitations related to the information on ADHD diagnosis from the NPR. The youngest children in MoBa were born in 2009 and we have information on ADHD diagnosis in MoBa up to 2017 in our data file, hence the youngest children are 8 years old in the NPR sample. As children, especially girls, might receive an ADHD diagnosis at an age older than 8, there might be some false negatives in the sample, which has been found in the comparative pregnancy cohort in Denmark [81]. In addition, a recent study investigating the practice of diagnosing ADHD in children in Norway found that only about half of the diagnoses were properly documented in the medical records, with inadequate differential diagnostic assessment being the main reason for unsatisfactory documentation for the ADHD diagnosis [82].

## Conclusion

In this paper we found that higher overall maternal diet quality and lower consumption of ultra-processed foods in pregnancy were associated with lower child ADHD symptom scores, but the association estimates were small. For child ADHD diagnosis, lower risk was found only for higher overall maternal diet quality measure. We found no associations of child diet quality at 3 years with either outcome.

We emphasize that no inferences regarding causation should be made based on these results, as unmeasured confounding could contribute to the observed associations. Also, as this is the first study investigating maternal and child diet quality in relation to both child ADHD symptom score and ADHD diagnosis, it is too early to draw firm conclusions about the associations we found. Instead we encourage more research conducted on this topic, with robust methodological approaches related to study design, variable definitions and statistical analyses, which will allow for better identification of possible causal relationships.

## Supplementary Information

**Additional file 1.** The Impact of a Community-Oriented Problem-Based Learning Curriculum Reform on the Quality of Primary Care Delivered by Gradua

**Additional file 2.** PDQI score calculation. Detailed description of the PDQI score components and calculations

**Additional file 3.** Supplementary Table. Foods and beverages from group 4 in the NOVA classification included in the Ultra-Processed Food Index (UPFI)

**Additional file 4.** CDQI score calculation. Detailed description of the CDQI score components and calculations

**Additional file 5.** Supplementary figure. Visualization of covariate selection for maternal diet quality during pregnancy as exposure via a Directed Acyclic Graph

**Additional file 6.** Supplementary figure. Visualization of covariate selection for child diet quality at 3 years as exposure via a Directed Acyclic Graph.

**Additional file 7.** Supplementary table. Absolute AME and corresponding relative AME change in ADHD symptoms for one SD increase in PDQI, UPFI and CDQI score

**Additional file 8.** Supplementary table. Relative risk for ADHD diagnosis for one SD increase in PDQI, UPFI and CDQI score

**Additional file 9.** Supplementary figure. Relative AME (%) change in ADHD symptom score for one SD increase in PDQI, UPFI and CDQI score, stratified by child sex and maternal ADHD symptoms (0–3, higher score equaling more symptoms)

**Additional file 10.** Supplementary figure. Relative risk for ADHD diagnosis for one SD increase in PDQI, UPFI and CDQI score, stratified by child sex and maternal ADHD symptoms (0–3, higher score equaling more symptoms)

## Data Availability

The dataset used for analyses in the current study is owned by the MoBa study and restrictions apply to the availability of these data. Researchers can apply for access to and use of data and biological samples from the MoBa in their research, and data access guidelines can be found here: https://www.fhi.no/en/studies/moba/for-forskere-artikler/research-and-data-access/. R-scripts used for analyses can be made available upon request to the corresponding author.

## Abbreviations

ADHD: Attention Deficit Hyperactivity Disorder
AME: Average marginal effect
CDQI: Child diet quality index
CI: Credible interval
FFQ: Food frequency questionnaire
MoBa: The Norwegian Mother, Father and Child Cohort Study
PDQI: Prenatal diet quality index
SD: Standard deviation
UPFI: Ultra-processed food index
WHO: World Health Organization
