# Supplementary material for: The associations between maternal and child diet quality and child ADHD – findings from a large Norwegian pregnancy cohort study
Source: BMC Psychiatry. 2021 Mar 8;21:139. doi: 10.1186/s12888-021-03130-4 (PMC7941947; doi:10.1186/s12888-021-03130-4)
Supplement: Supplementary file 1 — Additional file 1. The Impact of a Community-Oriented Problem-Based Learning Curriculum Reform on the Quality of Primary Care Delivered by Gradua [file 12888_2021_3130_MOESM1_ESM.pdf]

STROBE Statement—Checklist of items that should be included in reports of ***cohort studies***

	Item No	Recommendation	Section/ page/paragraph in manuscript
**Title and abstract**	1	(*a*) Indicate the study's design with a commonly used term in the title or the abstract	Title / p1
		(*b*) Provide in the abstract an informative and balanced summary of what was done and what was found	Abstract/ p2 -p3
**Introduction**			
Background/ rationale	2	Explain the scientific background and rationale for the investigation being reported	Background/ p4/ 1st paragraph - p5/2nd paragraph
Objectives	3	State specific objectives, including any prespecified hypotheses	Background/ p5/ 3rd paragraph
**Methods**			
Study design	4	Present key elements of study design early in the paper	Study population and design/ p6/2nd paragraph
Setting	5	Describe the setting, locations, and relevant dates, including periods of recruitment, exposure, follow-up, and data collection	Study population and design/ p6/ 2nd paragraph
Participants	6	(*a*) Give the eligibility criteria, and the sources and methods of selection of participants. Describe methods of follow-up	Study population and design/ p6/ 2nd paragraph and Figure 1
		(*b*) For matched studies, give matching criteria and number of exposed and unexposed	N/A
Variables	7	Clearly define all outcomes, exposures, predictors, potential confounders, and effect modifiers. Give diagnostic criteria, if applicable	Exposure definition/ p7/ 1st paragraph – covariates/ p11/ 3rd paragraph
Data sources/ measurement	8*	For each variable of interest, give sources of data and details of methods of assessment (measurement). Describe comparability of assessment methods if there is more than one group	Exposure definition/ p7/ 1st paragraph – covariates/ p11/ 3rd paragraph
Bias	9	Describe any efforts to address potential sources of bias	Statistical analyses/ p13/ 4th paragraph
Study size	10	Explain how the study size was arrived at	Study population and design/ p6/ 2nd paragraph and Figure 1
Quantitative variables	11	Explain how quantitative variables were handled in the analyses. If applicable, describe which groupings were chosen and why	Statistical analyses/ p12/ 3rd paragraph
Statistical methods	12	(*a*) Describe all statistical methods, including those used to control for confounding	Statistical analyses/ p12/ 3rd paragraph – Statistical analyses/ p13/ 3rd paragraph
		(*b*) Describe any methods used to examine subgroups and interactions	Statistical analyses/ p13/ 1st paragraph
		(*c*) Explain how missing data were addressed	Statistical analyses/ p13/ 4th paragraph - p14/ 1st paragraph
		(*d*) If applicable, explain how loss to follow-up was addressed	Statistical analyses/ p13/ 4th paragraph - p14/ 1st paragraph
		(*e*) Describe any sensitivity analyses	N/A
**Results**			
Participants	13*	(a) Report numbers of individuals at each stage of study—eg numbers potentially eligible, examined for eligibility, confirmed eligible, included in the study, completing follow-up, and analysed	Study population and design/ Figure 1
		(b) Give reasons for non-participation at each stage	Study population and design/

			Figure 1
		(c) Consider use of a flow diagram	Study population and design/ Figure 1
Descriptive data	14*	(a) Give characteristics of study participants (eg demographic, clinical, social) and information on exposures and potential confounders	Main results/ p15/ 2nd paragraph – Main results/ p16/ Table 3
		(b) Indicate number of participants with missing data for each variable of interest	Main results/ p16/ Table 3
		(c) Summarise follow-up time (eg, average and total amount)	Study population and design/ p6/ 2nd paragraph
Outcome data	15*	Report numbers of outcome events or summary measures over time	Main results/ p16/ Table 3
Main results	16	(*a*) Give unadjusted estimates and, if applicable, confounder-adjusted estimates and their precision (eg, 95% confidence interval). Make clear which confounders were adjusted for and why they were included	Main results/ p17/ 1st paragraph – p18/1st paragraph, Figure 2-4, Supplementary Table 3
		(*b*) Report category boundaries when continuous variables were categorized	N/A
		(*c*) If relevant, consider translating estimates of relative risk into absolute risk for a meaningful time period	Main results/ p18/1st paragraph and Figure 4
Other analyses	17	Report other analyses done—eg analyses of subgroups and interactions, and sensitivity analyses	Main results/ p17/ 2nd paragraph
**Discussion**			
Key results	18	Summarise key results with reference to study objectives	Discussion/ p18/2nd paragraph
Limitations	19	Discuss limitations of the study, taking into account sources of potential bias or imprecision. Discuss both direction and magnitude of any potential bias	Strengths and limitations/ p21/ 4th paragraph – p22/ 3rd paragraph
Interpretation	20	Give a cautious overall interpretation of results considering objectives, limitations, multiplicity of analyses, results from similar studies, and other relevant evidence	Discussion/ p18/3rd paragraph – p21/2nd paragraph
Generalisability	21	Discuss the generalisability (external validity) of the study results	Discussion/ p22/2nd paragraph
**Other information**			
Funding	22	Give the source of funding and the role of the funders for the present study and, if applicable, for the original study on which the present article is based	Funding/ p24/ 3rd paragraph

*Give information separately for exposed and unexposed groups.

Note: An Explanation and Elaboration article discusses each checklist item and gives methodological background and published examples of transparent reporting. The STROBE checklist is best used in conjunction with this article (freely available on the Web sites of PLoS Medicine at http://www.plosmedicine.org/, Annals of Internal Medicine at http://www.annals.org/, and Epidemiology at http://www.epidem.com/). Information on the STROBE Initiative is available at http://www.strobe-statement.org.
