# Supplementary material for: The associations between maternal and child diet quality and child ADHD – findings from a large Norwegian pregnancy cohort study
Source: BMC Psychiatry. 2021 Mar 8;21:139. doi: 10.1186/s12888-021-03130-4 (PMC7941947; doi:10.1186/s12888-021-03130-4)
Supplement: Supplementary file 2 — Additional file 2. PDQI score calculation. Detailed description of the PDQI score components and calculations [file 12888_2021_3130_MOESM2_ESM.pdf]

PDQI score calculation

We calculated the PDQI score using the method put forward by von Ruesten et al. [1] and modified it to correspond to the updated Norwegian food-based dietary guidelines [2]. The PDQI consists of 13 components, with a maximum score of ten per component (with the exception of total fish and fatty fish), for a maximum PDQI score of 120. We used different formulae to calculate scores depending on the intake recommendation for each included food group/nutrient:

1) For food groups with a minimum intake recommendation (fruit/berries, vegetables, and whole-grain, dairy): $PDQI\ score = \left(\frac{Reported\ intake}{Recommended\ intake}\right) \times 10$. For intakes above recommended minimum intake, we allocated a maximum score of 10.
2) For food groups and nutrients with a maximum intake recommendation (red meat, saturated fat, trans-fat, added sugar, salt): $PDQI\ score = \left(\frac{Recommended\ intake}{Reported\ intake}\right) \times 10$. For intakes below recommended maximum intake, we allocated a maximum score of 10.
3) For food groups with both a minimum and maximum recommendation (total fish and fatty fish) we used the formulae in 1) and 2) for intakes below the lower recommended intake level and above the upper recommended intake level, respectively. As to not overemphasize fish intake in the total PDQI score (as fatty fish is part of the total fish component), we divided the scores for total fish and fatty fish by two, with a possible maximum score of 5 for each component. For intakes within the recommended intake range, we allocated the maximum score.

Diet diversity

To reflect each participant's diet variation on a food level we included diet diversity as an additional component in the PDQI. There are recommendations regarding diet variation in the Norwegian food-based dietary guidelines, which justify inclusion of these components.

The diet diversity component is based on the diet diversity component from the revised Diet Quality Index (DQI-R) [3] and we follow the score calculation put forward by the DQI-R authors. This component reflects the intake variation of 25 food categories, depicted in table 2, with a higher score equaling greater diversity. To get a positive score for each food category, the participant had to have eaten a ¼ serving of that respective food per day, with an overall maximum diet diversity score of 10.

Supplementary Table. Diet Diversity Component Overview

Food groups	Representative foods	¼ portion (grams)
**Grains**		
Non-whole-grain breads	Low fiber bread, crispbread and biscuits	10
Non-whole-grain cereals	Low fiber cereals	13
Non-whole-grain crackers	Low fiber crispbread and biscuits	2
Pasta	All pasta dishes	50
Whole-grain breads	High fiber bread and crispbread	11
Whole-grain crispbread	High fiber crispbread	2.5
Whole-grain cereals	High fiber muesli and porridge	33
Rice	Rice and couscous	43
**Vegetables**		
Other vegetables	Alliums, mushrooms, peppers, cucumber, celery, squash, corn, tomato, peas, avocado	25
White potatoes	Mashed and fried potatoes, French fries	30
Nuts	Almonds, peanuts, other nuts	13
Legumes	Lentils, kidney beans, soy products	56
Root vegetables	Swede, carrot	25
Cabbage vegetables	Cauliflower, broccoli, cabbage, brussels sprouts	25
Dark green and leafy vegetables	Spinach, lettuce	25
**Fruits**		
Citrus fruit, melons, berries	Orange, grapefruit, all berries	25
All other fruits and juices	Apples, bananas, grapes, raisins, pear, mango, papaya, plums	25
**Animal components**		
Red meat	Beef, pork, lamb, offal	34-71
Milk	Plain milk, chocolate milk	50
Game	Reindeer, moose, wild boar	38
Poultry	Chicken, turkey	38
Cheese	White cheese, brown cheese, blue cheese	5
Eggs	Eggs, seagull eggs	14
Fish	Seafood, lean fish, fatty fish, fish roe, fish fingers	11
Yogurt	Yoghurt, probiotic milk	31

References

1. von Ruesten, A., et al., *Adherence of pregnant women to Nordic dietary guidelines in relation to postpartum weight retention: results from the Norwegian Mother and Child Cohort Study*. BMC Public Health, 2014. **14**: p. 75.
2. Norwegian Directorate of Health, *Norwegian guidelines on diet, nutrition and physical activity [in Norwegian]*. 2014: Oslo, Norway.
3. Haines, P.S., A.M. Siega-Riz, and B.M. Popkin, *The Diet Quality Index Revised.* Journal of the Academy of Nutrition and Dietetics, 1999. **99**(6): p. 697-704.
