# Supplementary material for: The associations between maternal and child diet quality and child ADHD – findings from a large Norwegian pregnancy cohort study
Source: BMC Psychiatry. 2021 Mar 8;21:139. doi: 10.1186/s12888-021-03130-4 (PMC7941947; doi:10.1186/s12888-021-03130-4)
Supplement: Supplementary file 3 — Additional file 3. Supplementary Table. Foods and beverages from group 4 in the NOVA classification included in the Ultra-Processed Food Index (UPFI) [file 12888_2021_3130_MOESM3_ESM.pdf]

Supplementary Table: Foods and beverages from group 4 in the NOVA classification included in the Ultra-Processed Food Index (UPFI)

| Food and beverage groups |
|---|
| **Alcoholic beverages** |
| **Bacon** |
| **Biscuits** |
| **Cereals with added sugars** |
| **Drinks with artificial sweeteners** |
| **Fish sticks** |
| **French fries** |
| **Hamburgers** |
| **Ice creams** |
| **Lunch meats** |
| **Margarines** |
| **Meat spreads** |
| **Pastries** |
| **Pizza** |
| **Powdered soups and sauces** |
| **Sausages** |
| **Savoury snacks** |
| **Store bought condiments** |
| **Sugar added to foods/beverages** |
| **Sugary spreads** |
| **Sweets/candies** |
| **White bread** |
