# Supplementary material for: The associations between maternal and child diet quality and child ADHD – findings from a large Norwegian pregnancy cohort study
Source: BMC Psychiatry. 2021 Mar 8;21:139. doi: 10.1186/s12888-021-03130-4 (PMC7941947; doi:10.1186/s12888-021-03130-4)
Supplement: Supplementary file 4 — Additional file 4. CDQI score calculation. Detailed description of the CDQI score components and calculations [file 12888_2021_3130_MOESM4_ESM.pdf]

CDQI score calculation

Diet diversity

The diet diversity component reflects the intake variation in the foods consumed by the child. In the original DQI, this component included all FG's as well as sweets/snacks in the score calculation. We modified this and included only foods/beverages that are categorized into one of the main food groups. To receive a score for each FG, the child has to have consumed at least one serving of each respective food per day from each of the main FG's. The diet diversity score is denoted as a percentage with possible range of 0% - 100% and is calculated with the following formulae:

Diet diversity score = $\left(\frac{\text{Recommended intake, no.of FG's}}{\text{Reported intake, no.of FG's}}\right) \times 100$

Diet quality

The diet quality of a food or beverage depend on its nutrient- and energy density, and in this component the foods are categorized as follows;

- the preference group (e.g. fresh fruit, vegetables and whole grains); each food is factored by 1
- the moderation group (e.g. white bread); each food is factored by 0
- the "low nutrient, energy dense" group (e.g. soft drinks and snacks); each food is factored by -1

The diet quality score is denoted as a percentage with possible range of -100% - 100%, with higher percentage equalling better diet quality, and is calculated with the following formulae:

Diet quality score = $\left(\frac{\text{Food intake quantity x factor of food}}{\text{Total quantity all FG's}}\right)$

Diet equilibrium: adequacy and moderation

The diet equilibrium component introduces the concept of dietary balance to the CDQI, taking into account both "adequacy" and "moderation": adequate intake of foods in the preferred group, like fruits, vegetables and whole grain products, combined with intake moderation, with particular focus on limiting the intake of low nutrient energy dense foods. The calculation of the dietary equilibrium score follows three steps, is denoted as percentage with

possible range of 0 - 100%, with higher percentage equalling better dietary balance, and is calculated with the following formulae:

$$\text{Diet adequacy score} = \frac{\left(\frac{\text{Reported intake within each FG}}{\text{Minimum recommendations for FG}}\right)}{\text{Total number of FG's}} \times 100$$

$$\text{Diet moderation score} = \frac{\left(1-\left(\frac{\text{Reported intake within each FG} - \text{upper recommendations for FG}}{\text{Upper recommendations for FG}}\right)\right)}{\text{Total number of FG's}} \times 100$$

$$\text{Diet equilibrium score} = \frac{\left(\frac{\text{Dietary adequacy for FG}}{\text{Dietary excess for FG}}\right)}{\text{Total number of FG's}} \times 100$$

$$\text{Final CDQI score} = \left(\frac{\text{Diet diversity score} + \text{diet quality score} + \text{diet equilibrium score}}{3}\right)$$
