# Supplementary material for: The associations between maternal and child diet quality and child ADHD – findings from a large Norwegian pregnancy cohort study
Source: BMC Psychiatry. 2021 Mar 8;21:139. doi: 10.1186/s12888-021-03130-4 (PMC7941947; doi:10.1186/s12888-021-03130-4)
Supplement: Supplementary file 5 — Additional file 5. Supplementary figure. Visualization of covariate selection for maternal diet quality during pregnancy as exposure via a Directed Acyclic Graph [file 12888_2021_3130_MOESM5_ESM.pdf]

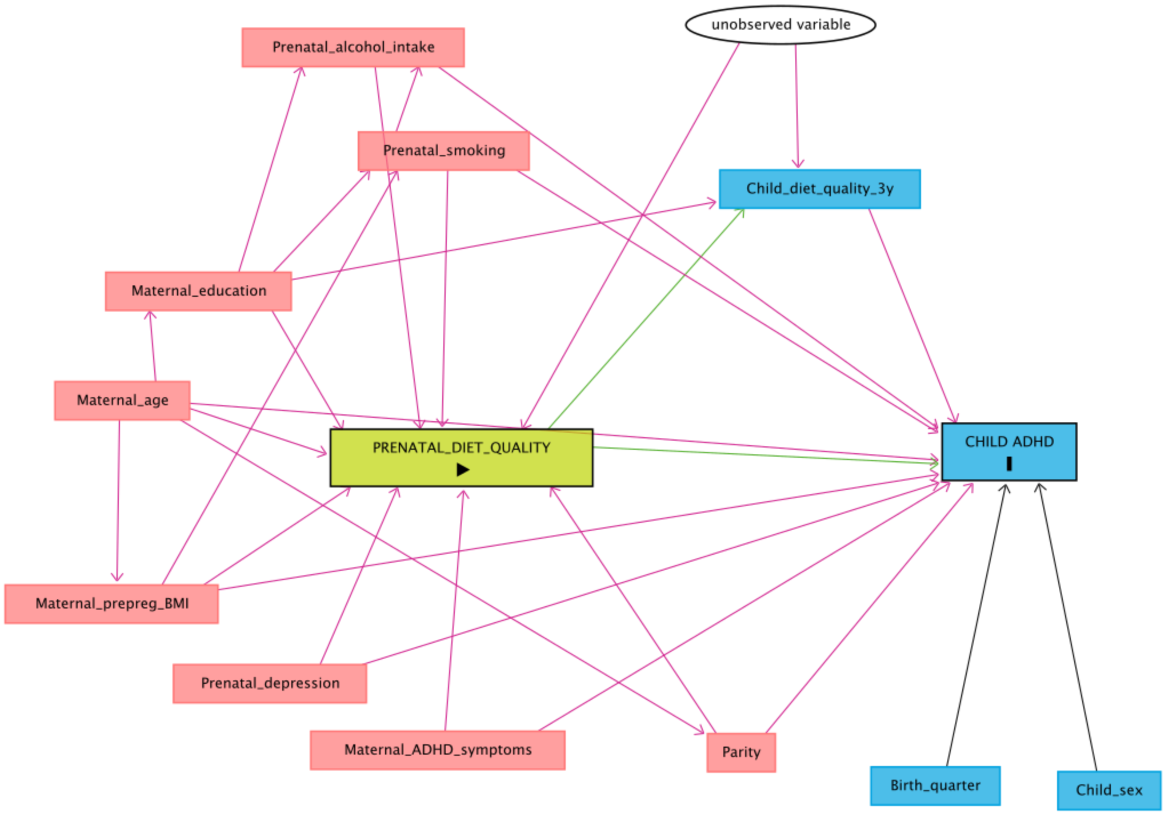

Supplementary Figure: Visualization of covariate selection for prenatal diet quality as exposure via a Directed Acyclic Graph
