# Supplementary material for: The associations between maternal and child diet quality and child ADHD – findings from a large Norwegian pregnancy cohort study
Source: BMC Psychiatry. 2021 Mar 8;21:139. doi: 10.1186/s12888-021-03130-4 (PMC7941947; doi:10.1186/s12888-021-03130-4)
Supplement: Supplementary file 6 — Additional file 6. Supplementary figure. Visualization of covariate selection for child diet quality at 3 years as exposure via a Directed Acyclic Graph. [file 12888_2021_3130_MOESM6_ESM.pdf]

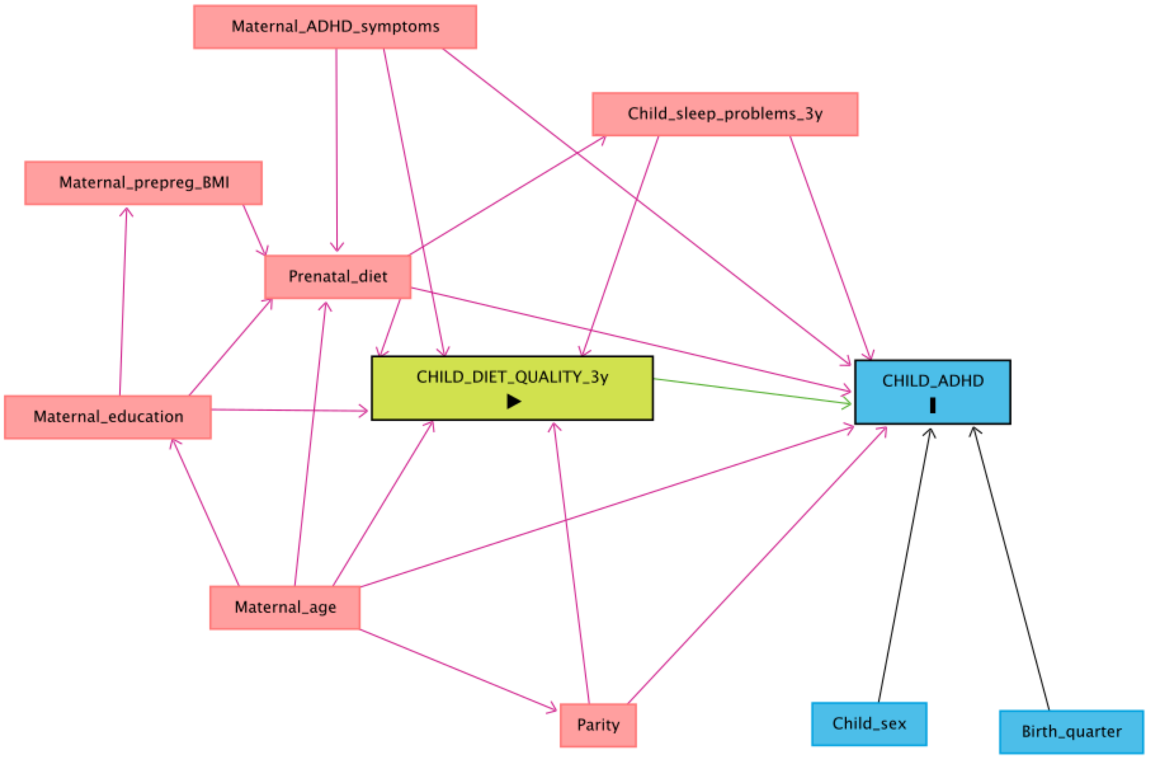

Supplementary Figure: Visualization of covariate selection for postnatal diet quality as exposure via a Directed Acyclic Graph
