# Supplementary material for: The associations between maternal and child diet quality and child ADHD – findings from a large Norwegian pregnancy cohort study
Source: BMC Psychiatry. 2021 Mar 8;21:139. doi: 10.1186/s12888-021-03130-4 (PMC7941947; doi:10.1186/s12888-021-03130-4)
Supplement: Supplementary file 7 — Additional file 7. Supplementary table. Absolute AME and corresponding relative AME change in ADHD symptoms for one SD increase in PDQI, UPFI and CDQI score [file 12888_2021_3130_MOESM7_ESM.pdf]

Supplementary Table: Absolute AME and corresponding relative AME change in ADHD symptoms for one SD increase in PDQI, UPFI and CDQI score

Diet quality indices	ADHD symptom score							
	Crude model[1]				Adjusted model[2]			
	Absolute AME	CI	Relative AME	CI	Absolute AME	CI	Relative AME	CI
**PDQI**	-0.45	-0.55, -0.34	-5.3%	-6.5%, -4.0%	-0.28	-0.41, -0.14	-3.3%	-4.8%, -1.6%
**UPFI**	0.38	0.27, 0.49	4.5%	3.3%, 5.9%	0.25	0.13, 0.38	3.0%	1.5%, 4.5%
**DQI3y**	-0.22	-0.35, -0.10	-2.6%	-4.2%, -1.2%	-0.06	-0.18, 0.06	-0.7%	-2.2%, 0.7%

Adjustment variables: None

[2] Adjustment variables: For PDQI and UPFI: maternal pre-pregnancy BMI, maternal education, smoking and alcohol intake during pregnancy, maternal symptoms of depression and ADHD, maternal age, parity, child sex, child diet and child birth quarter. For CDQI: maternal pre-pregnancy BMI, maternal education, maternal symptoms of ADHD, maternal age, prenatal diet quality, child sex, parity, child sleep problems (3y) and child birth quarter.
