# Supplementary material for: The associations between maternal and child diet quality and child ADHD – findings from a large Norwegian pregnancy cohort study
Source: BMC Psychiatry. 2021 Mar 8;21:139. doi: 10.1186/s12888-021-03130-4 (PMC7941947; doi:10.1186/s12888-021-03130-4)
Supplement: Supplementary file 8 — Additional file 8. Supplementary table. Relative risk for ADHD diagnosis for one SD increase in PDQI, UPFI and CDQI score [file 12888_2021_3130_MOESM8_ESM.pdf]

Supplementary Table: Relative risk for ADHD diagnosis for one SD increase in PDQI, UPFI and CDQI score

Diet quality indices	ADHD diagnosis			
	Crude model[1]		Adjusted model[2]	
	Relative Risk	CI	Relative Risk	CI
**PDQI**	0.78	0.73, 0.84	0.87	0.79, 0.97
**UPFI**	1.00	0.93, 1.08	1.07	0.99, 1.18
**DQI3y**	0.87	0.79, 0.93	0.99	0.90, 1.08

[1] Adjustment variables: None
[2] Adjustment variables: For PDQI and UPFI: maternal pre-pregnancy BMI, maternal education, smoking and alcohol intake during pregnancy, maternal symptoms of depression and ADHD, maternal age, parity, child sex, child diet and child birth quarter. For CDQI: maternal pre-pregnancy BMI, maternal education, maternal symptoms of ADHD, maternal age, prenatal diet quality, child sex, parity, child sleep problems (3y) and child birth quarter.
