# Supplementary material for: The associations between maternal and child diet quality and child ADHD – findings from a large Norwegian pregnancy cohort study
Source: BMC Psychiatry. 2021 Mar 8;21:139. doi: 10.1186/s12888-021-03130-4 (PMC7941947; doi:10.1186/s12888-021-03130-4)
Supplement: Supplementary file 9 — Additional file 9. Supplementary figure. Relative AME (%) change in ADHD symptom score for one SD increase in PDQI, UPFI and CDQI score, stratified by child sex and maternal ADHD symptoms (0–3, higher score equaling more symptoms) [file 12888_2021_3130_MOESM9_ESM.pdf]

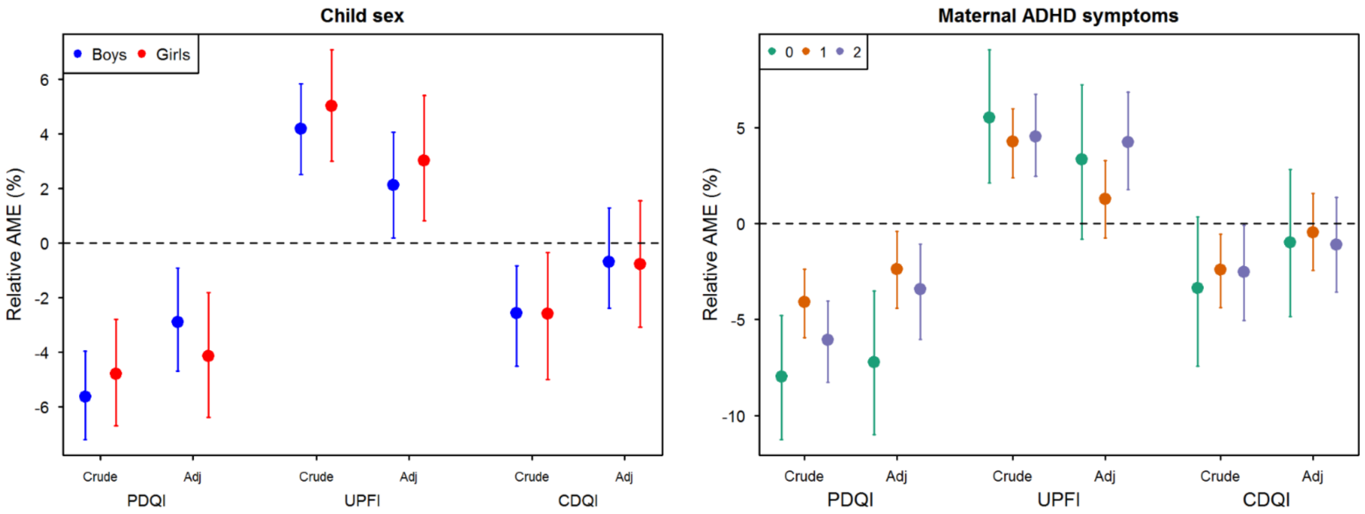

Supplementary Figure: Relative AME (%) change in ADHD symptom score for one SD increase in PDQI, UPFI and CDQI score, stratified by child sex and maternal ADHD symptoms (0-3, higher score equaling more symptoms)
