# Supplementary material for: The associations between maternal and child diet quality and child ADHD – findings from a large Norwegian pregnancy cohort study
Source: BMC Psychiatry. 2021 Mar 8;21:139. doi: 10.1186/s12888-021-03130-4 (PMC7941947; doi:10.1186/s12888-021-03130-4)
Supplement: Supplementary file 10 — Additional file 10. Supplementary figure. Relative risk for ADHD diagnosis for one SD increase in PDQI, UPFI and CDQI score, stratified by child sex and maternal ADHD symptoms (0–3, higher score equaling more symptoms) [file 12888_2021_3130_MOESM10_ESM.pdf]

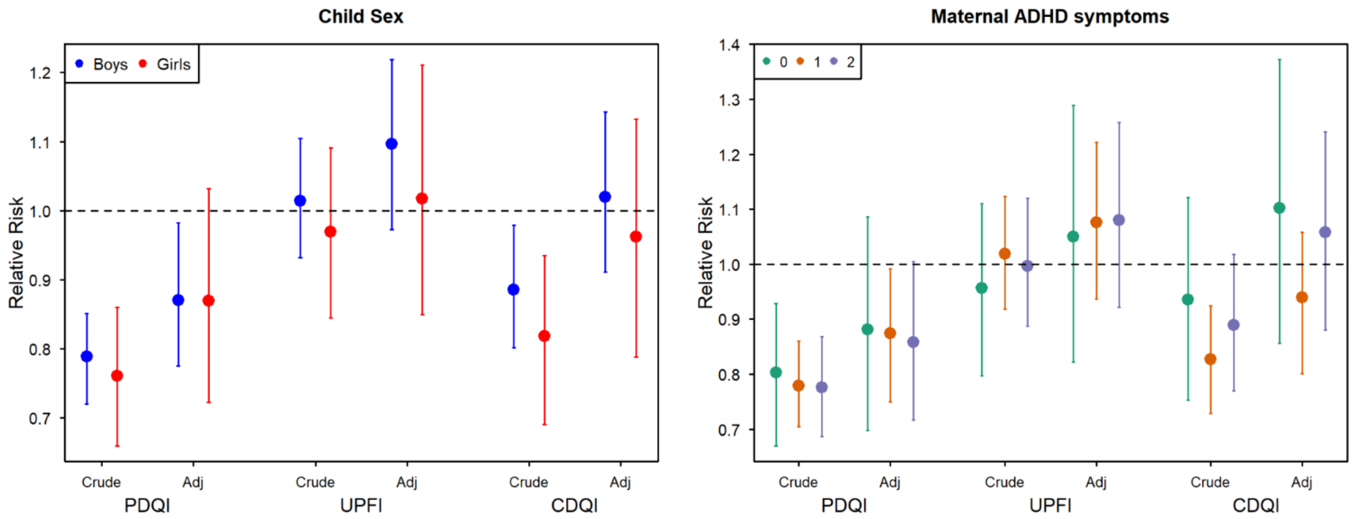

Supplementary Figure: Relative risk for ADHD diagnosis for one SD increase in PDQI, UPFI and CDQI score, stratified by child sex and maternal ADHD symptoms (0-3, higher score equaling more symptoms)
